# Single-cell Atlas reveals core function of CPVL/MSR1 expressing macrophages in the prognosis of triple-negative breast cancer

**DOI:** 10.3389/fimmu.2024.1501009

**Published:** 2024-12-24

**Authors:** Xinan Wang, Li Lin, Xue Zhang, Minghui Zhang, Zhuo Sun, Yichen Yang, Xiuna Zhang, Yonghui Yuan, Yong Zhang, Hao Chen, Ti Wen

**Affiliations:** ^1^ Department of Medical Oncology, The First Hospital of China Medical University, Shenyang, Liaoning, China; ^2^ Key Laboratory of Precision Diagnosis and Treatment of Gastrointestinal Tumors, Ministry of Education, The First Hospital of China Medical University, Shenyang, Liaoning, China; ^3^ Key Laboratory of Anticancer Drugs and Biotherapy of Liaoning Province, The First Hospital of China Medical University, Shenyang, Liaoning, China; ^4^ Clinical Cancer Treatment and Research Center of Shenyang, The First Hospital of China Medical University, Shenyang, Liaoning, China; ^5^ Department of Pulmonary and Critical Care Medicine, Institute of Respiratory Disease, The First Hospital of China Medical University, Shenyang, Liaoning, China; ^6^ Department of Gynecology, The First Hospital of China Medical University, Shenyang, Liaoning, China; ^7^ Department of Medical Oncology, Second People’s Hospital of Huludao, Huludao, Liaoning, China; ^8^ Cancer Hospital of Dalian University of Technology, Liaoning Cancer Hospital & Institute, Shenyang, Liaoning, China; ^9^ Department of Pathology, Cancer Hospital of Dalian University of Technology, Liaoning Cancer Hospital & Institute, Shenyang, Liaoning, China; ^10^ Department of Breast Surgery, The First Hospital of China Medical University, Shenyang, Liaoning, China

**Keywords:** single-cell sequence, macrophages, cPVL, MSR1, prognosis, triple-negative breast cancer

## Abstract

**Background:**

Triple-negative breast cancer (TNBC) is the most aggressive subtype of breast cancer, with the worst prognosis among all subtypes. The impact of distinct cell subpopulations within the tumor microenvironment (TME) on TNBC patient prognosis has yet to be clarified.

**Methods:**

Utilizing single-cell RNA sequencing (scRNA-seq) integrated with bulk RNA sequencing (bulk RNA-seq), we applied Cox regression models to compute hazard ratios, and cross-validated prognostic scoring using a GLMNET-based Cox model. Cell communication analysis was used to elucidate the potential mechanisms of CPVL and MSR1. Ultimately, RNA interference-mediated gene knockdown was utilized to validate the impact of specific genes on the polarization of tumor-associated macrophages (TAMs).

**Results:**

Our findings revealed that the function of immune cells is more pivotal in prognosis, with TAMs showing the strongest correlation with TNBC patient outcomes, compared with other immune cells. Additionally, we identified CPVL and MSR1 as critical prognostic genes within TAMs, with CPVL expression positively correlated with favorable outcomes and MSR1 expression associated with poorer prognosis. Mechanistically, CPVL may contribute to favorable prognosis by inhibiting the SPP1-CD44 ligand-receptor and promoting CXCL9-CXCR3, C3-C3AR1 ligand-receptor, through which TAMs interact with other cells such as monocytes, neutrophils, and T cells. Moreover, cytokines including IL-18, IFNγR1, CCL20, and CCL2, along with complement-related gene like TREM2 and complement component CFD, may participate in the process of CPVL or MSR1 regulating macrophage polarization. Furthermore, RT-PCR experiments confirmed that CPVL is positively associated with M1-like TAM polarization, while MSR1 is linked to M2-like TAM polarization. Finally, the prognostic significance of these two genes is also validated in HER2-positive breast cancer subtypes.

**Conclusions:**

CPVL and MSR1 are potential biomarkers for macrophage-mediated TNBC prognosis, suggesting the therapeutic potential of macrophage targeting in TNBC.

## 1 Introduction

Breast cancer is a highly heterogeneous disease that can be classified into several subtypes based on molecular characteristics. Among them, triple-negative breast cancer (TNBC) is responsible for 10-15% of all breast cancers but accounts for 40% of breast cancer-related deaths worldwide (1). Due to the lack of estrogen receptor (ER), progesterone receptor (PR), and human epidermal growth factor receptor 2 (HER2) expression, as well as the absence of effective therapeutic targets and treatment options, TNBC is considered a subtype with poor outcomes. The prognosis of TNBC is closely linked to its tumor microenvironment (TME). Aside from the tumor cells themselves, various cellular components in the TME can regulate tumor development through complex crosstalk, ultimately influencing patient prognosis. At present, algorithms developed using bulk RNA data, such as CIBERSORT, have been used to identify specific cell types within the TME, including T cells (2), B cells (3), NK cells (4) and tumor-associated macrophages (TAMs) (5), as linked to the prognosis of TNBC patients. However, the complex roles of individual cells in TME remain to be discovered. With the advancement of single-cell RNA sequencing (scRNA-seq) technology, the analysis of the TME has reached unprecedented precision. This technique offers higher resolution and reduced error compared to earlier methods such as pathology, immunohistochemistry, and bulk RNA sequencing. However, since single-cell sequencing data lack clinical information and prognostic data, it is challenging to conduct large-scale comparisons like those possible with bulk sequencing, making it difficult to analyze the prognostic influence of different cell subtypes or assess the function of key genes within these subtypes in disease prognosis or therapeutic efficacy.

Prior investigations have reported that macrophage subpopulations were highly prevalent in the tumor immune microenvironment (TIME) of TNBC (5), and their polarization state was a key factor influencing prognosis. In TNBC, M1-like polarized macrophages are associated with favorable prognosis (5), whereas M2-like macrophages, typically considered TAMs, are linked to poor prognosis (6). Moreover, macrophage polarization status and the expression of polarization-specific genes exhibit heterogeneity across different cancer types. For instance, TREM2 is positively correlated with M1-like macrophages in cervical squamous cell carcinoma and endometrial adenocarcinoma but is associated with M2-like macrophages in lung squamous cell carcinoma, clear cell renal carcinoma, and invasive ductal carcinoma of the breast (7). In lung cancer, NLRP6 (8) and HHLA2 (9), in glioma, IGFBP2 (10), and in TNBC, COL5A1 (11), MCT-1 (12), and Sohlh2 (13) promote M2-like polarization of macrophages. Although many macrophage polarization-related genes have been identified in previous TNBC studies, those most strongly associated with patient prognosis remain to be discovered.

Here, we identified CPVL and MSR1 as key genes in TAMs within the TNBC TME, both of which significantly influence patient prognosis. CPVL and MSR1 were found to be associated with macrophage polarization. CPVL encodes a serine carboxypeptidase that is primarily expressed in macrophages and monocytes (14), located in the endoplasmic reticulum and lysosomal compartments. In previous studies, the relationship between CPVL and macrophages in tumors has only been demonstrated in gastric cancer, where it is positively correlated with M2-like macrophage polarization, contributing to poor patient prognosis (15). MSR1, commonly known as a macrophage-specific gene, encodes the macrophage scavenger receptor 1, a trimeric integral membrane glycoprotein involved in various functional and disease-related mechanisms related to macrophages, including Alzheimer’s disease, atherosclerosis, and host defense (16). Additionally, MSR1 induces M2 macrophage polarization through the regulation of proline and arginine metabolism (17), and copy number variations (CNVs) in MSR1 may influence the risk of developing several types of cancer (18). Although previous studies have demonstrated that CPVL and MSR1 were expressed in macrophages and affected macrophage-related physiological and pathological processes, their prognostic significance in TNBC has not been previously established. In this study, we demonstrated that CPVL and MSR1 are specifically expressed in TAMs and are significantly correlated with TNBC prognosis. By using CellChat analysis to examine cell communication and performing correlation analysis between genes, cytokines, and components of the complement pathway, we explored the potential regulatory mechanisms of these genes.

In this study, to perform parallel comparisons of different cell subpopulations within the TME at the single-cell level, we analyzed 66 TNBC tissues and integrated data from the TCGA and GEO databases, comprising 230 TNBC patients with prognostic information. By combining scRNA-seq with bulk RNA sequencing (bulk RNA-seq), we highlighted that, compared to other cell types, macrophages were the most prognostically significant cells in the TME of TNBC, with CPVL and MSR1 being key prognostic genes. Targeting these genes may provide new avenues for developing strategies to improve outcomes in TNBC patients.

## 2 Materials and methods

### 2.1 Data source and preprocessing

For the scRNA-seq dataset, original data were downloaded from GEO, including GSE255107, GSE180286, GSE176078, GSE118389, GSE199515, GSE246613, and GSE161529, comprising a total of 66 samples (265477 cells). Quality control of the scRNA-seq data was performed using the “Seurat” package: cells with fewer than 300 genes or more than 6,500 genes expressed, or those with >10% mitochondrial gene expression, were excluded. For the bulk RNA-seq dataset, we downloaded the breast cancer BRCA dataset from the TCGA database and the GSE103091 breast cancer dataset from GEO. Mean centering the genes were performed on each dataset to correct for batch effects and platform differences. The cohorts were then combined, and patients who were ER, PR, or HER2 positive were excluded, leaving a total of 230 patients for further analysis.

### 2.2 Single-cell sequencing analysis

The “SCTransform” function was used to integrate the normalized data to correct for platform-specific biases. Then, the data were scaled and subjected to principal component analysis (PCA). The “Harmony” package in R was used to remove batch effects from the segregated scRNA-seq data. With “principal components” = 20, TNBC cell cluster analysis was performed using “FindNeighbors” and “FindCluster” functions. The uniform manifold approximation and projection (UMAP) method was used to visualize the data. Differentially expressed genes between the clusters were identified and manually annotated based on known biomarkers for each cell type. Markers used in this study are listed: lymphocytes (CD20, CD3E), myeloid cells (CD14, CSF3R), endothelial cells (PECAM1, CDH5), fibroblasts (THY1, PDGFRB), tumor cells (EPCAM, TP63), T cells (CD3D), B cells (MS4A1), neutrophils (FCGR3B), monocytes (FCN1), mast cells (KIT), macrophages (C1QB), dendritic cells (CD1E). To further investigate, tumor-associated macrophages were classified into four groups based on the average expression of CPVL and MSR1 in macrophages.

### 2.3 Differential gene identification and mapping to bulk data

Dominant genes expressed in each major cell type were identified in the scRNA-seq dataset (i.e., genes with a fold change >3 relative to other cell types, *P*-value <0.05). These genes were then projected onto bulk RNA-seq data to shows their relative expression pattern. Spearman’s correlation coefficient was used to calculate the correlation between the expression levels of each gene, and a heatmap was generated.

### 2.4 Cox regression hazard analysis

According to bulk RNA-seq data and clinical information, hazard ratios (95% confidence interval, displayed as horizontal bars with *P*-values) were obtained using a multivariate Cox regression model, and cross-validated prognostic scores were generated using a GLMNET-based Cox model and applied to pairwise differences in gene expression. A factor was considered an independent prognostic feature if the *P*-value was less than 0.05 in both univariate and multivariate Cox analyses.

### 2.5 Survival analysis of prognostic genes

To identify survival-related genes within a given cell type, cell type-specific prognostic feature analysis was performed. Based on the aforementioned prognostic scores and hazard ratios, seven genes within macrophages that significantly influenced prognosis (*P* < 0.05) in the bulk dataset of 230 TNBC patients were identified. Kaplan-Meier survival curves were generated using the median prognostic score as a cutoff to differentiate genes into “Better Genes” and “Worse Genes” based on their opposing effects on prognosis. The expression of these seven prognostic genes was then further analyzed in relation to patient survival.

### 2.6 Linear correlation analysis

Linear fitting of single-cell data was performed using lm(). Gene set enrichment analysis was conducted on each cell type using the fgsea R package, and gene-gene correlations were calculated using Spearman’s correlation coefficients. Scatter plots were created using the ggplot2 R package. *P*-values were computed using the t-distribution, and correlation fitting curves with 95% confidence intervals were drawn when *P*-values were less than 0.05.

### 2.7 Cell-cell communication analysis

We employed the CellChat R package (version 1.6.1) (https://github.com/sqjin/CellChat) to study intercellular communication and identify signaling molecules at the single-cell level. First, we processed gene expression data to pinpoint ligands and receptors highly expressed within individual cell clusters. Next, we evaluated cell-cell communication at the pathway level by calculating the communication probability for all ligand-receptor interactions associated with each signaling pathway. These probabilities were then aggregated to construct an intercellular communication network.

### 2.8 Correlation analysis between CPVL and MSR1 gene expression and pathway scores, cytokine, and complement gene expression

To explore the potential functional pathways of the genes, we retrieved HALLMARK pathways from https://www.gsea-msigdb.org/. The AddModuleScore function was used to compute the HALLMARK pathway scores for each cell. Cytokine-related genes were obtained from the KEGG_CYTOKINE_CYTOKINE_RECEPTOR_INTERACTION path way, and complement-related genes were acquired from the GOBP_COMPLEMENT_ACTIVATION pathway. Spearman’s correlation coefficients were calculated between gene expression and pathway scores, as well as between genes and cytokine/complement genes, using the psych R package. The results were visualized using the ggcorrplot R package.

### 2.9 Cell culture

Human THP-1 monocytes were kindly supplied by Ya-jing Fu from the First Affiliated Hospital of China Medical University. Cells were cultured under sterile conditions in RPMI-1640 medium supplemented with 15% fetal bovine serum, 1% penicillin (100 U/mL), and 1% streptomycin (100 μg/mL). The cells were passaged every 2-3 days. All cell cultures were maintained in a cell incubator at 37°C with 5% CO_2_ and saturated humidity. For experiments, cells in the logarithmic growth phase were used, and mycoplasma contamination was regularly checked via quantitative PCR to ensure negative results.

### 2.10 Cell transfection

All siRNAs were from JTS scientific. CPVL siRNA was used to transfect THP-1 M0 cells at a confluency of 70%-90% using jetPRIME, according to the manufacturer’s instructions. At 48 to 72 hours post-transfection, RNA was extracted from the cells, and qPCR analysis was performed. The sequence of siRNA targeting CPVL is: siRNA1: 5’-CGGCUUCCUCACCGUGAAUTT-3’, 5’-AUUCACGGUGAGGAAGC CGTT-3’, siRNA2: 5’-CUACUAGAUGGCGACUUAATT-3’, 5’-UUAAGUCG CCAUCUAGUAGTT-3’.

### 2.11 *In vitro* differentiation of THP-1 cells

A total of 2.5 × 10^5^ THP-1 cells were plated in culture dishes and pretreated with 100 ng/mL of PMA, then transfected with siRNA. After 24 hours, the medium was changed, and cells were divided into two groups: one group was treated with 100 ng/mL LPS and IFN-γ to induce M1-like macrophage differentiation, and the other group was treated with 50 ng/mL IL-4 to induce M2-like macrophage differentiation. Polarization was confirmed by detecting cell surface markers using qPCR.

### 2.12 RNA extraction and qRT-PCR

Cell samples from different treatment conditions were collected. Total RNA was extracted using the Eastep Super Total RNA Extraction Kit (Promega, LS1040) according to the manufacturer’s instructions. The extracted RNA was quantified by measuring absorbance at 260 nm using a NanoDrop ND-100 spectrophotometer. Reverse transcription was performed using the GoScript Reverse Transcription Kit (Promega, A2790) to synthesize cDNA from the RNA. Real-time quantitative PCR (qRT-PCR) was conducted using SYBR Premix Ex Taq II on an Applied Biosystems 7500 Real-Time PCR System. The qRT-PCR protocol consisted of 45 cycles of 50°C for 2 minutes, 95°C for 10 minutes, 95°C for 15 seconds, and 60°C for 1 minute, followed by a final cycle of 95°C for 15 seconds, 60°C for 1 minute, 95°C for 30 seconds, and 60°C for 15 seconds. Relative expression levels were calculated using the 2-ΔΔCt method, with 18S as the internal control. The primer sequences were as follows: CPVL forward primer: 5’-TGACCTTGCGTGACAGAGAC-3’, CPVL reverse primer: 5’-CCGTGCACCGCAAAAAGTTA-3’; MSR1 forward primer: 5’-GCCAACCTCATGGACACAGA-3’, MSR1 reverse primer: 5’-AGAATTTCCTGGCCTTCCGG-3’; CD163 forward primer: 5’-GAAGACAGAGACAGCGGCTT-3’, CD163 reverse primer: 5’-GGTATCTTAAAGGCTCACTGGGT-3’; CD86 forward primer: 5’-CACACGGATGAGTGGGGTC-3’, CD86 reverse primer: 5’-ACTGAAGTTAGCAGAGAGCAGG-3’; 18s forward primer: 5’-CCCGGGGAGGTAGTGACGAAAAAT-3’, 18s reverse primer: 5’-CGCCCGCCCGCTCCCAAGAT-3’ (Sangon Biotech (Shanghai) Co., Ltd.).

### 2.13 Immune infiltration analysis

CIBERSORT, a deconvolution method, was used to analyze the degree of cellular immune infiltration based on the bulk datasets. In this study, multivariate Cox regression analysis was used to assess the impact of CM ratio, TNM stage, and macrophage infiltration on patient prognosis in each breast cancer subtype.

### 2.14 Statistical analysis

All statistical tests, regression fitting, and plotting in this study were performed using R statistical software (https://www.R-project.org). Spearman’s rank correlation was applied for all correlation analyses. Kaplan-Meier analysis was used to generate survival curves for each prognostic gene in the dataset. Unless otherwise specified (such as in dominant marker gene analyses), all statistical tests were two-sided, and a *P*-value < 0.05 was considered statistically significant. Appropriate multiple testing corrections were applied where necessary, as described in each analysis section.

## 3 Results

### 3.1 Immune cell compartment has most significant impact on the prognosis of TNBC patients

**Figure 1:**
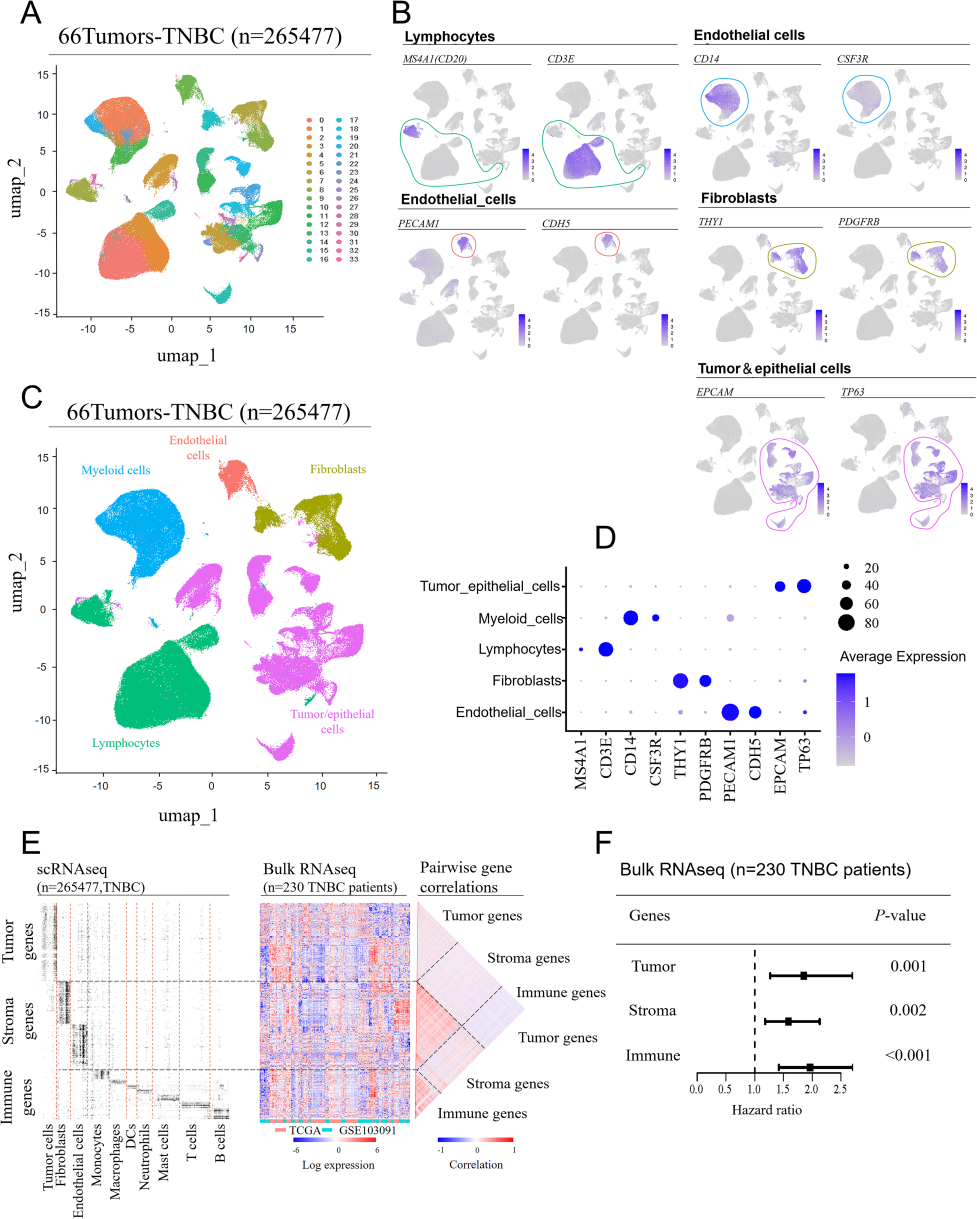
Cell compartment of the tumor microenvironment in TNBC patients. Single-cell RNA sequencing (scRNA-seq) data from seven GEO datasets were integrated, covering 66 tissue samples (batch effects were removed using the “Seurat” package in R). **(A)** UMAP was used to separate the cell clusters. **(B)** Manual annotation was performed based on the expression characteristics of marker genes for the five cell cluster. **(C)** UMAP visualization of single-cell transcriptomes from 66 TNBC samples, showing the separation of major cell lineages. **(D)** A bubble plot displaying the expression levels of marker genes across different cell types. **(E)** Identification of dominant genes in each major cell type based on scRNA-seq data (fold-change > 3 compared to other cell types; adjusted *P*-value < 0.05) (left). These genes were mapped onto a bulk RNA-seq dataset from 230 TNBC patients (middle), showing their relative expression patterns, and pairwise correlations between the same genes (right). **(F)** Comparison of the prognostic impact of tumor-, stromal-, and immune-related genes. Hazard ratios for each feature were obtained using a multivariate Cox regression model (wald 95% confidence intervals and *P*-values are shown as horizontal bars), with cross-validated prognostic scores calculated using a GLMNET-based Cox model.

To investigate the most prognostically significant cellular components within the TME of TNBC, we integrated scRNA-seq data from 66 TNBC patient tissue samples, encompassing 265,477 single-cell transcriptomes. Using unsupervised clustering and UMAP, we categorized the cells into 33 distinct cell clusters (**Figure 1A**), which reflect the complexity of the TME in TNBC. Each cluster was annotated based on known marker genes for specific cell types (**Figure 1B**): clusters 0, 2, 3, 7, 15, and 29 predominantly expressed lymphocyte markers, clusters 1, 10, 20, 22, 24, and 32 expressed myeloid cell markers, clusters 9 and 33 expressed endothelial cell markers, clusters 6, 8, and 17 expressed fibroblast markers, and clusters 4, 5, 11, 12, 13, 14, 16, 18, 19, 21, 23, 25, 26, 27, 28, 30, and 31 predominantly expressed tumor/epithelial cell markers. The tumor cell group contained more clusters than other groups, which indicates that breast cancer cells have the most heterogeneity. Subsequently, the single-cell transcriptomes from 66 TNBC samples were visualized by clear separation between the major cell types, including fibroblasts, endothelial cells, tumor/epithelial cells, lymphocytes, and myeloid cells (**Figure 1C**). Additionally, a bubble plot demonstrated the expression intensity of marker genes in each cell type, validating the annotations of these cell populations (**Figure 1D**).

Next, we classified the identified cell subpopulations into three major compartments: tumor cells (tumor/epithelial cells), immune cells (myeloid cells, lymphocytes) and stromal cells (endothelial cells, fibroblasts). The expression levels of dominant genes in each cell type (tumor cells, fibroblasts, endothelial cells, *etc.*) were visualized using a heatmap in the scRNA-seq dataset (**Figure 1E**, left). Since scRNA-seq data lacked prognostic information, we then mapped these dominant genes onto a bulk RNA sequencing dataset from a 230 TNBC patients’ cohort, displaying them in another heatmap (**Figure 1E**, center). Eventually, a pairwise correlation analysis (**Figure 1E**, right) revealed that genes specific to each cell compartment (tumor, stromal, immune) were enriched within their respective compartment, suggesting that coregulation patterns observed in bulk mRNA datasets are largely driven by differences in cell-type abundance across the TME.

Based on the analyses above, we further explored the impact of the three major cell compartments—tumor cells, immune cells, and stromal cells — on clinical outcomes of TNBC. By mapping the feature genes of these three major cell types to the bulk RNA-seq data from 230 TNBC patients with prognostic information, we obtained hazard ratios using a multivariate Cox regression model, combined with cross-validated prognostic scores based on a GLMNET Cox model. The analysis revealed that feature genes from all three cell compartments — tumor genes (*P* = 0.001), stromal genes (*P* = 0.002), and immune genes (*P* < 0.001) — were significantly associated with clinical outcomes, with immune genes showing the strongest correlation with prognosis in TNBC patients (**Figure 1F**). Given that immune feature genes are enriched in immune cells, it can be concluded that immune cells have a significantly greater effect on the prognosis of TNBC patients.

### 3.2 Macrophages are the most prognostically significant immune cell subpopulation in TNBC

**Figure 2:**
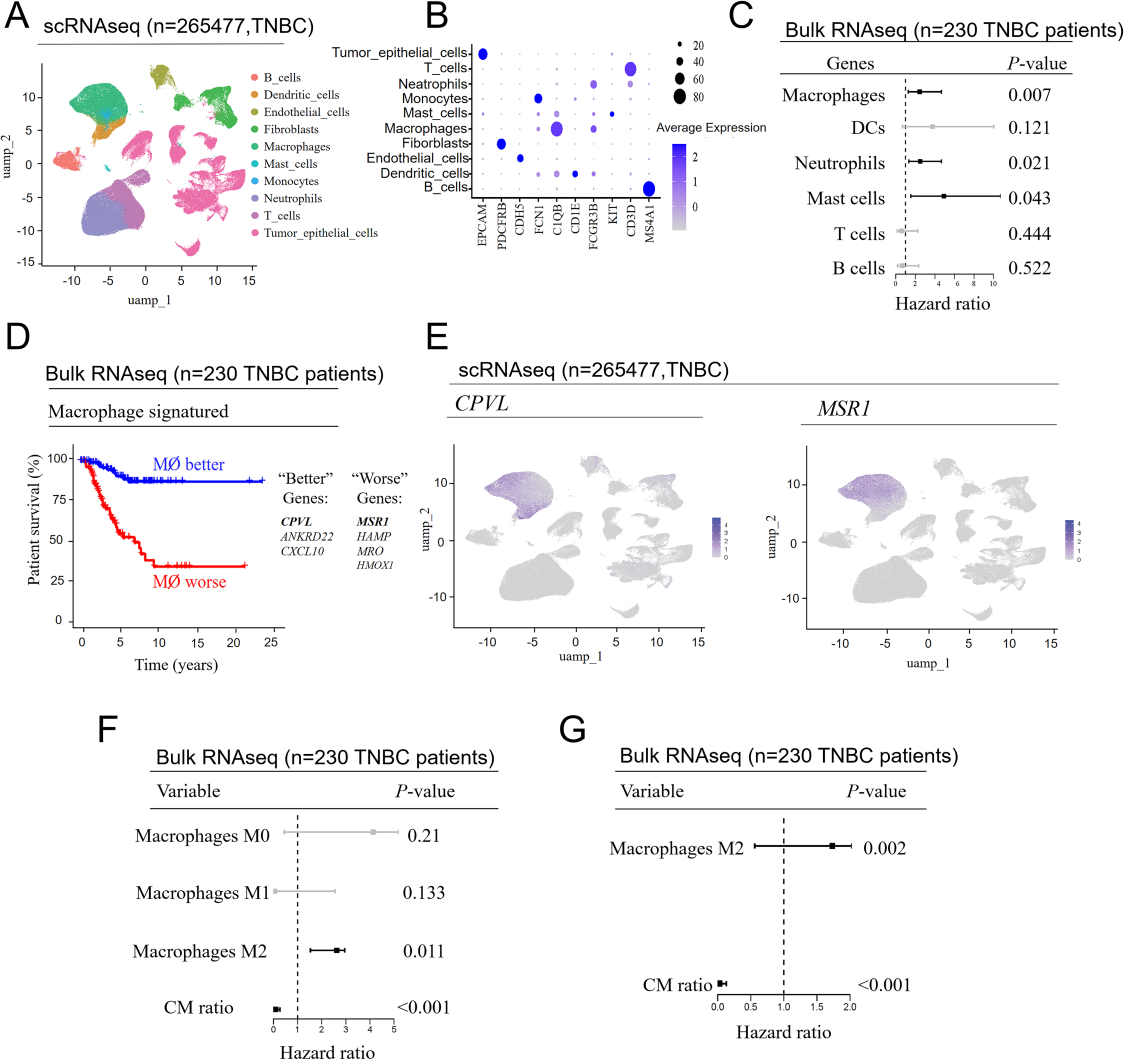
CPVL and MSR1 in macrophages are genes significantly associated with the prognosis of TNBC patients. **(A)** UMAP plot of immune cells, showing further subclustering of the cell populations. **(B)** Bubble plot illustrating the expression patterns of marker genes in the different immune cell subpopulations. **(C)** Repeated analysis from the image **(A)**, highlighting the impact of different immune cell subpopulations on prognosis. **(D)** Cross-validated macrophage prognostic scores from the image **(C)** were used to generate Kaplan-Meier curves, showing genes with opposite effects on prognosis at the 50% cutoff. **(E)** Display of the primary expression situation of CPVL and MSR1 in macrophages. **(F)** The degree of macrophage infiltration in patients’ tumors was first calculated using “CIBERSORT”, followed by the application of a univariate Cox model to assess the impact of macrophage infiltration and the CPVL/MSR1 ratio (CM ratio) on patient prognosis. **(G)** A multivariate Cox model was employed to assess the impact of M2 macrophage infiltration and CM ratio on patient prognosis.

The immune cell cluster comprises multiple subpopulations with distinct functions, each exerting varying effects on prognosis. Firstly, we refined the immune cell subclusters from single-cell RNA sequencing data and visualized them using UMAP (**Figure 2A**). A dot plot (**Figure 2B**) illustrated the expression of marker genes in each immune cell subtype, further validating their annotations as B cells, T cells, dendritic cells, macrophages, mast cells, neutrophils, and monocytes. To determine which immune cell subpopulation was most strongly associated with patient prognosis, we mapped the feature genes of these immune subpopulations onto the bulk RNA-seq data. Multivariate Cox regression analysis was used to obtain hazard ratios, and cross-validated prognostic scores were calculated using a GLMNET Cox model. The analysis revealed that only three cell types—mast cells (*P* = 0.043), neutrophils (*P* = 0.021), and macrophages (*P* = 0.007)—were significantly associated with TNBC prognosis (**Figure 2C**). Based on the combined analysis of *P*-values and hazard ratios, macrophages emerged as the most prognostically significant immune cell subpopulation in TNBC.

### 3.3 CPVL and MSR1 are key genes in macrophages affecting TNBC prognosis

Recent studies showed that genes such as MCT-1, COL5A1, and Sohlh2 promoted M2-like macrophage polarization and were associated with poor prognosis in TNBC (11–13). Conversely, genes like IFI35 were linked to M1-like macrophage polarization and favorable outcomes in TNBC (5). To further explore the connection between gene expression in macrophages and prognosis in TNBC patients, we used cross-validated macrophage prognostic scores from bulk RNA-seq data and illustrated the opposing effects of selected genes at the 50% cutoff value using Kaplan-Meier survival curves (**Figure 2D**). Among the identified macrophage-related prognostic genes, CPVL, ANKRD22, and CXCL10 were positively correlated with favorable prognosis, while MSR1, HAMP, MRO, and HMOX1 were associated with poor prognosis. To validate the expression of these genes in macrophages, we visualized their distribution using global UMAP dimensionality reduction, confirming that CPVL was expressed in both macrophages and dendritic cells, whereas MSR1 was restricted to macrophages (**Figure 2E**). Although other genes are also restricted to macrophages, their expression levels are relatively low (**Supplementary Figures S1A–E**), suggesting that CPVL and MSR1 are more accurate indicators of macrophage-related prognosis.

Next, we used the CPVL/MSR1 ratio to compare the prognosis of patients with different macrophage polarization states via univariate Cox analysis. The analysis reveals that only M2-like macrophages (*P* = 0.011) and the CM ratio (*P* < 0.001) are significantly linked to prognosis (**Figure 2F**). Further multivariate analysis (**Figure 2G**) shows that M2-like macrophages are linked to poor prognosis, whereas the CM ratio is correlated with better outcomes, demonstrating that the CM ratio has a stronger independent prognostic effect.

**Figure 3:**
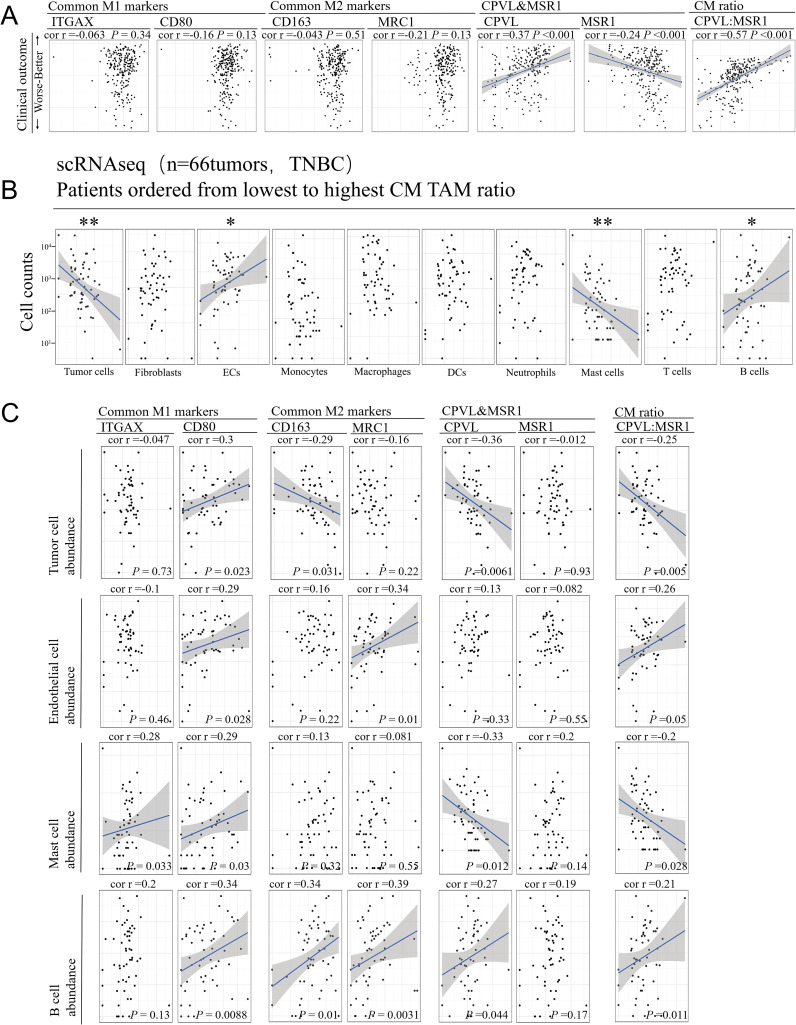
The coordinated relationship between CPVL/MSR1 and immune response in TNBC. **(A)** The scatter plot shows a lack of correlation between patient prognosis and common M1 and M2 markers, while there is a significant correlation with the individual expression of CPVL, MSR1, and the CM ratio. Spearman’s rank correlation was used, and a fitted blue line was shown when significant. **(B)** Cell counts of major cell types are displayed, and Spearman’s rank correlation analysis was used to assess correlations with CM ratio. **(C)** The correlation between the abundance of tumor cells, endothelial cells, mast cells, and B cells with common M1 and M2 markers, CPVL, MSR1, and CM ratio. Spearman’s rank correlation was used. *Indicates that the P value is less than 0.05. **Indicates that the P value is less than 0.01.

The effect of CPVL and MSR1 on the outcome of TNBC patients remains to be elucidated. Using Spearman’s rank correlation analysis, we demonstrate via scatter plot (**Figure 3A**) that high CPVL expression is associated with better clinical outcomes, while high MSR1 expression correlates with worse outcomes. Moreover, the CPVL/MSR1 ratio shows a stronger correlation with TNBC prognosis than individual gene expression, underscoring its significance. In contrast, despite their established roles in tumor development, the expression of M1 and M2 classic markers shows no significant association with clinical outcomes in this cohort, which suggests that the classic markers cannot be directly applied to predict prognosis. So it is essential to further explore the complex gene phenotypes of macrophages under specific disease conditions. The survival curves of the seven genes are shown in **Supplementary Figures S2A–E**.

It is important to note that apparent coregulation patterns in bulk RNA data are likely driven by the heterogeneity of cell subtype abundance in TME. Therefore, we performed Spearman’s rank correlation analysis based on CM ratio, finding that only four cell types in the single-cell RNA data from 66 TNBC tumor samples were correlated with CM ratio (**Figure 3B**): tumor cell and mast cell abundance were negatively correlated with CM ratio, while endothelial cell and B cell abundance were positively correlated. The CM ratio shows no correlation with other immune or stromal cell types, including tumor-associated macrophages themselves. This suggests a potential interaction between CPVL and MSR1 expression and these four cell types. Spearman’s correlation analysis (**Figure 3C**) reveals that the CM ratio has a stronger association with the abundance of these four cell types in TNBC patients than M1 or M2 markers. Overall, these findings indicate a coordinated relationship between TAMs and other cell types in TME, suggesting they may provide more insight into CPVL and MSR1 expression in TAM than traditional M1 and M2 markers.

### 3.4 Interactions between CPVL and MSR1-expressing TAMs and other cell subpopulations in the TNBC microenvironment

**Figure 4:**
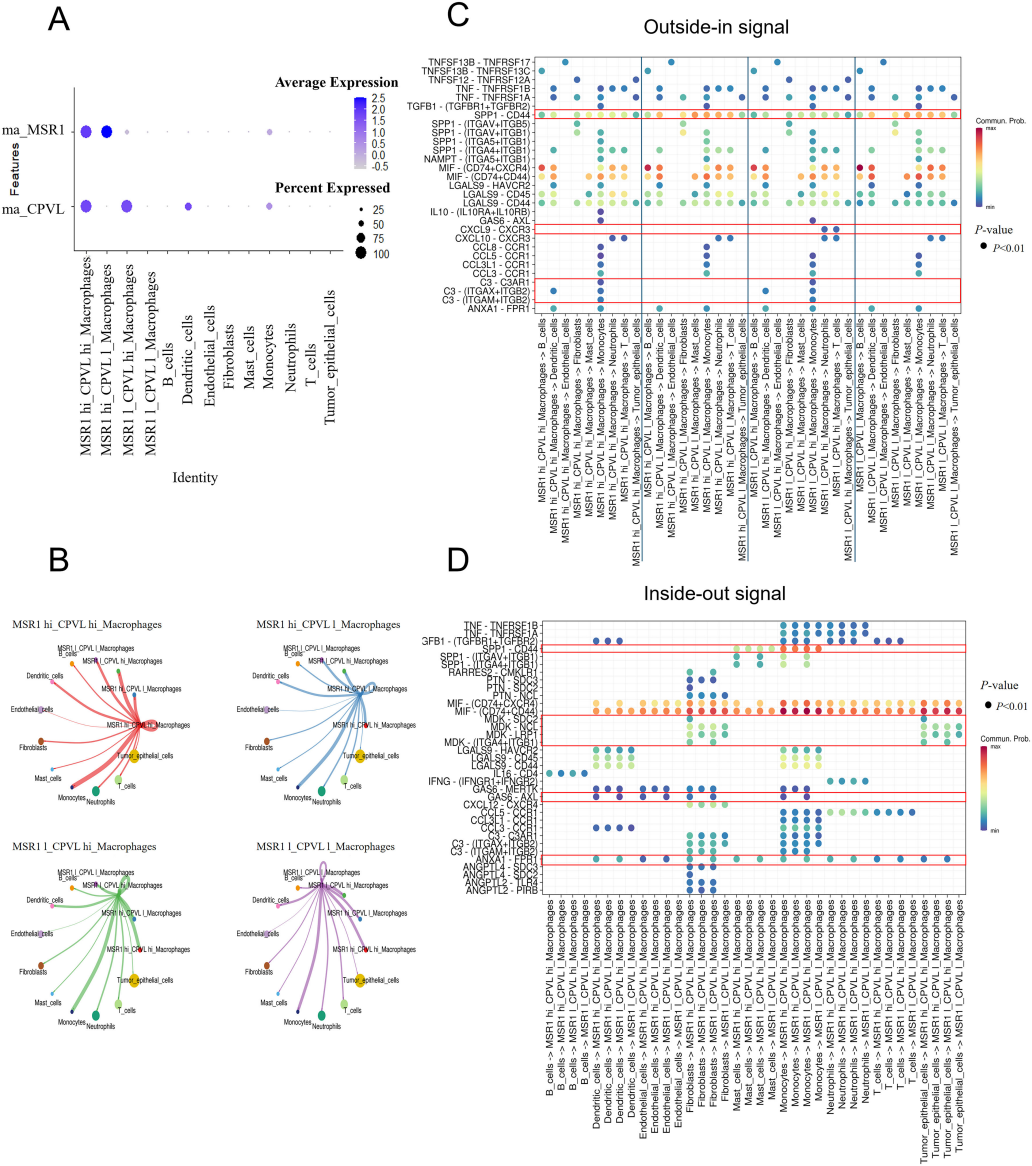
CPVL and MSR1 are involved in regulating signaling between macrophages and other cells. **(A)** A bubble plot displaying the expression of MSR1 and CPVL across the four macrophage subpopulations and other cell types. **(B)** CellChat analysis showing the cell-cell communication between the four macrophage subpopulations and other cells. **(C, D)** Dot plots illustrating ligand-receptor interactions between the four macrophage subpopulations and other cells.

To deeply explore the intercellular interactions and potential regulatory networks of CPVL- and MSR1-expressing TAMs within TME, we divided macrophages into four subgroups based on the average expression of MSR1 and CPVL: MSR1^hi^CPVL^hi^, MSR1^hi^CPVL^low^, MSR1^low^CPVL^hi^, and MSR1^low^CPVL^low^. A bubble plot showing the expression of MSR1 and CPVL across different TAMs (**Figure 4A**) confirmed, consistent with previous findings, that CPVL and MSR1 were primarily expressed in macrophages, with CPVL also found in dendritic cells. Using CellChat analysis, we found that these four macrophage groups had rich signaling interactions, both among themselves and with other cells (**Figure 4B**). Cells expressing high levels of CPVL and/or MSR1 show greater ability to interact with monocytes, while cells with lower CPVL and MSR1 expression have reduced interaction capacity, suggesting that CPVL and MSR1 facilitate intercellular communication between TAMs and monocytes. Previous studies have demonstrated that macrophage-monocyte interactions promoted the differentiation of monocytes into macrophages (19). Additionally, TAMs were found to interact not only with monocytes but also with dendritic cells, highlighting a potential mechanism by which TAMs contribute to the TNBC immune microenvironment through their interactions with myeloid cells.

Next, we explored the specific signaling pathways by which CPVL and MSR1 mediate interactions between TAMs and other cells (**Figures 4C, D**). CellChat analysis reveals ligand-receptor interactions between the four macrophage subgroups and other cells, showing that all four TAM groups received SPP1-CD44 signals from monocytes and mast cells. However, CPVL^low^ TAMs sent more SPP1-CD44 signals to other cell subpopulations than CPVL^hi^ TAMs. Studies have identified the SPP1 pathway as a crucial mediator of interaction between TAMs and tumor epithelial cells, and between TAMs themselves (20). SPP1^+^ macrophages can also interact with tumor-associated fibroblasts, epithelial cells, and malignant cells, promoting fibrosis (21), extracellular matrix restructuring, and the formation of an immune-suppressive tumor barrier (20, 22, 23). These processes have been linked to poor prognosis (24), suggesting that CPVL^low^ TAMs may promote tumor progression and the development of an immunosuppressive microenvironment. In contrast, CPVL^hi^ TAMs sent higher levels of C3 signals to monocytes, with multiple ligand-receptor pairs such as C3-(ITGAX+ITGB2), C3-(ITGAM+ITGB2) and C3-C3AR1, being involved. Complement protein C3a has been shown to induce pro-inflammatory (M1-like) macrophage polarization, while C3b promotes anti-inflammatory (M2-like) polarization (25). The regulation of the C3 signaling pathway by CPVL is complex, but the C3-C3AR1 pathway is clearly defined, stimulating the production of complement C3a, and triggering an inflammatory response. This indicates that CPVL^hi^ TAMs may stimulate monocytes to polarize into M1-like macrophages through C3a production. We also observed that only MSR1^low^CPVL^hi^ TAMs sent CXCL9-CXCR3 signals to neutrophils and T cells. Upregulation of the CXCL9-CXCR3 axis enhances T cell infiltration (26) and improves immune response (27), indicating that TAMs with a high CM ratio may play a key role in boosting immune responses and anti-tumor activity. Collectively, these signaling pathways may explain the positive prognostic impact of CPVL^hi^ macrophages.

Despite the positive correlation between CPVL expression in TAM and patients’ prognosis, CPVL^hi^ TAM signaling within the TNBC microenvironment remains complex. Compared to CPVL^low^ TAMs, CPVL^hi^ TAMs received more GAS6 signals from fibroblasts, endothelial cells and dendritic cells, also, more ANXA1 signals from neutrophils, monocytes, dendritic cells, fibroblasts, endothelial cells, and tumor cells. GAS6-AXL and ANXA1-FPR1 signaling regulated macrophage polarization and initiation of macrophages, promoting the M2-like macrophage phenotype within the TME (28–30). The specific mechanisms by which these signals affect CPVL^hi^ TAMs require further investigation. Additionally, MDK-SDC2 and MDK-(ITGA4+ITGB1) signals from fibroblasts and tumor cells were only received by CPVL^hi^ TAMs. MDK has been reported to promote M2-like macrophage polarization through the MDK-LRP1 axis in gallbladder cancer (31), leptomeningeal metastasis (32), and clear cell renal cell carcinoma (33), contributing to poor prognosis. However, the impact of MDK-SDC2 and MDK-(ITGA4+ITGB1) signaling on macrophages remains unexplored. The potential role of these signals in macrophage polarization and patient prognosis warrants further investigation. Overall, while survival curves indicate better outcomes for patients with high CPVL expression, these TAMs may still be under the regulation of negative signals. Therefore, CPVL-expressing TAMs may unleash anti-tumor effects by inhibiting SPP1-CD44 and promoting CXCL9-CXCR3 and C3-C3AR1 ligand-receptor reciprocal effects with other cells, like monocytes, neutrophils, and T cells. However, they may also promote tumor progression through GAS6-AXL and ANXA1-FPR1 signaling, interacting with fibroblasts, endothelial cells, and monocytes, while the role of MDK-SDC2 and MDK-(ITGA4+ITGB1) signals remains to be elucidated.

### 3.5 Potential regulatory mechanisms of CPVL and MSR1 in the TNBC microenvironment

**Figure 5:**
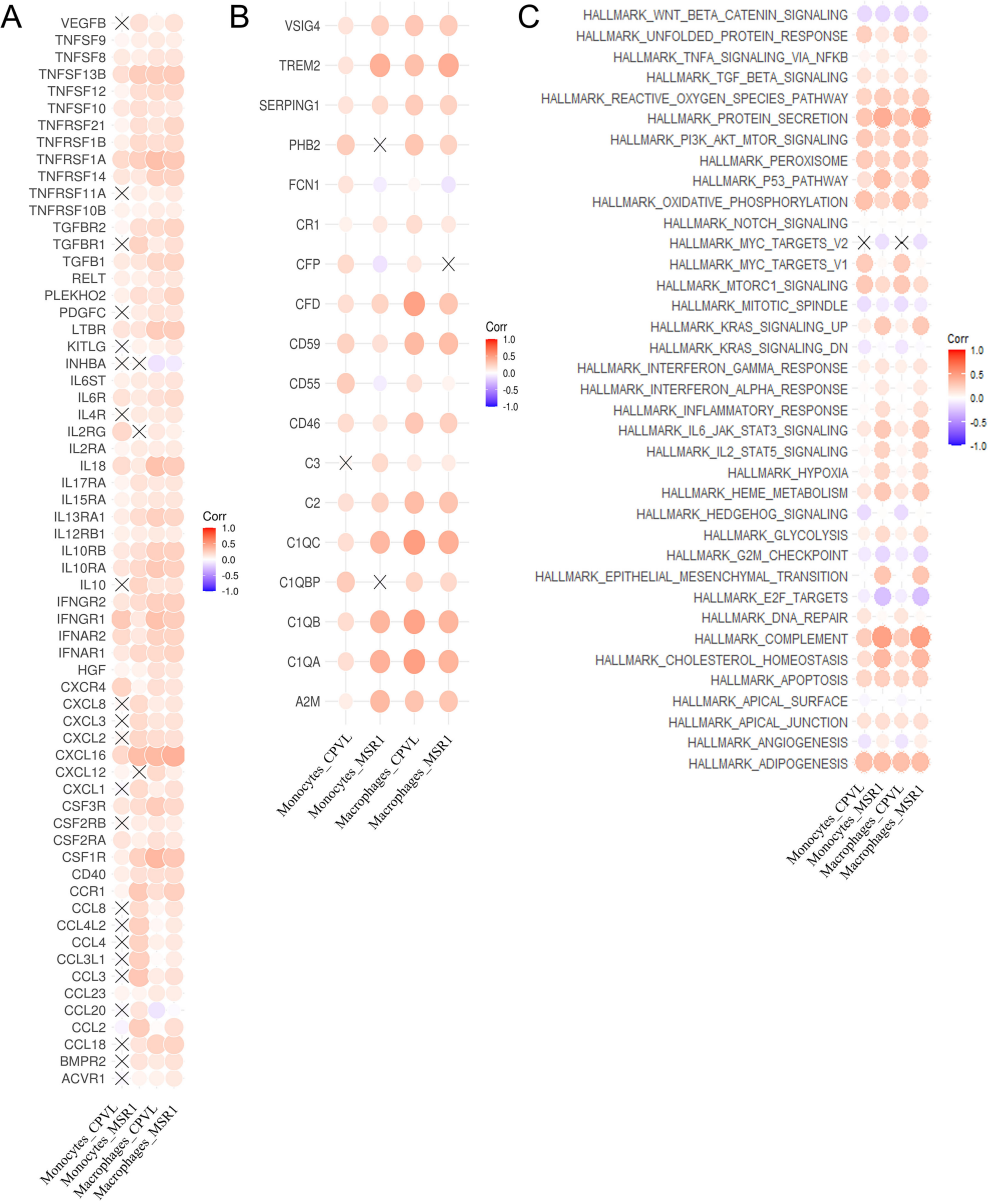
Regulatory effects of CPVL and MSR1 on expression of cytokines, complement, and pathways. **(A)** The correlation between the expression of cytokines and MSR1 or CPVL in monocytes and macrophages. **(B)** The correlation between the expression of complement-related and MSR1 or CPVL in monocytes and macrophages. **(C)** The correlation between the HALLMARK pathway scores and MSR1 or CPVL in monocytes and macrophages.

Macrophages that have received signals produce various cytokines to mediate immune response in the TME. To probe the cytokines involved in CPVL^+^ and/or MSR1^+^ TAMs function, we analyzed the correlation between CPVL and MSR1 expression and cytokine gene expression in monocytes and macrophages (**Figure 5A**). IL-18 and IFNγR1 showed significantly higher expression levels in CPVL^+^ TAMs compared to MSR1^+^ TAMs, and both genes were more strongly expressed in macrophages than in monocytes. Previous studies have shown that IL-18 stimulated Th1 and NK cells and induced IFN-γ production in macrophages (34), driving M1-like polarization (27). This suggests that CPVL^+^ TAMs may exhibit autocrine activation effects and contribute to promoting immune responses within the TME.

In contrast, the expression of the CCL20 gene was positively associated with MSR1^+^ TAMs and negatively associated with CPVL^+^ TAMs. CCL20 has been shown to promote CD163 expression in macrophages and induce TAM infiltration as well as M2-like macrophage polarization in tumor tissues (35, 36). This reveals that CCL20 may contribute to the immunosuppressive effects of MSR1^+^ TAMs in the TNBC microenvironment, correlating with poor prognosis, whereas CPVL TAMs may have the opposite effect. Additionally, CCL2 was highly expressed in MSR1^+^ monocytes and TAMs in TNBC patients but not in CPVL^+^ cells. CCL2 has been found to promote EMT processes and enhance cancer stem cell characteristics in TNBC patients (37), as well as induce M2-like polarization in resident macrophages (38). This suggests that CCL2 may drive M2-like polarization in MSR1^+^ TAMs within TNBC breast tissue, contributing to poor outcomes. Therefore, TAMs with a low CM ratio may promote tumor progression and immune suppression by upregulating CCL20 and CCL2, which is a potential cause of poor patient prognosis.

Next, we examined the correlation between CPVL and MSR1 expression and complement-related genes in monocytes and macrophages (**Figure 5B**). We found that TREM2 was more highly expressed in MSR1^+^ cells than in CPVL^+^ cells. TREM2 in macrophages has recently been shown to reduce CD8^+^ T cell anti-tumor activity (35) and promotes an immunosuppressive environment in breast cancer (39), which correlates with poor outcomes (40). It has also been found to regulate M2 polarization in other diseases, such as osteoarthritis and lung cancer (7, 41). This suggests that MSR1^+^ TAMs may promote the transition from M1- to M2-like macrophages through TREM2 expression, while inhibiting T cell activity. Additionally, CFD gene expression is positively correlated only with CPVL^+^ TAMs. CFD activated the complement pathway, amplifying complement-mediated bactericidal effects and enhancing the phagocytic activity of inflammatory macrophages (42). In summary, the findings indicate that the regulation of cytokines and complement components may explain why patients with a high CM ratio have better prognosis, providing potential new strategies for improving TNBC treatment outcomes.

Finally, we analyzed the correlation between CPVL and MSR1 expression and HALLMARK pathways’ scores in monocytes and macrophages (**Figure 5C**). In MSR1^hi^ macrophages, the KRAS, EMT, ANGIOGENESIS, HYPOXIA, and COMPLEMENT pathways were activated. These pathways have been implicated in angiogenesis (43), the induction of M2-like polarization in macrophages (44, 45), and tumor metastasis (46), suggesting that MSR1 may promote angiogenesis and metastasis in TNBC, contributing to poor prognosis. In CPVL^hi^ macrophages, the MYC-Targets-V1 pathway was activated, which is associated with high mutational burden, metastatic breast cancer invasion, and poor prognosis (47). This indicates that CPVL may also participate in the MYC-Targets-V1 pathway, negatively influencing patient prognosis.

### 3.6 The role of CPVL and MSR1 in macrophage polarization and other breast cancer subtypes

**Figure 6:**
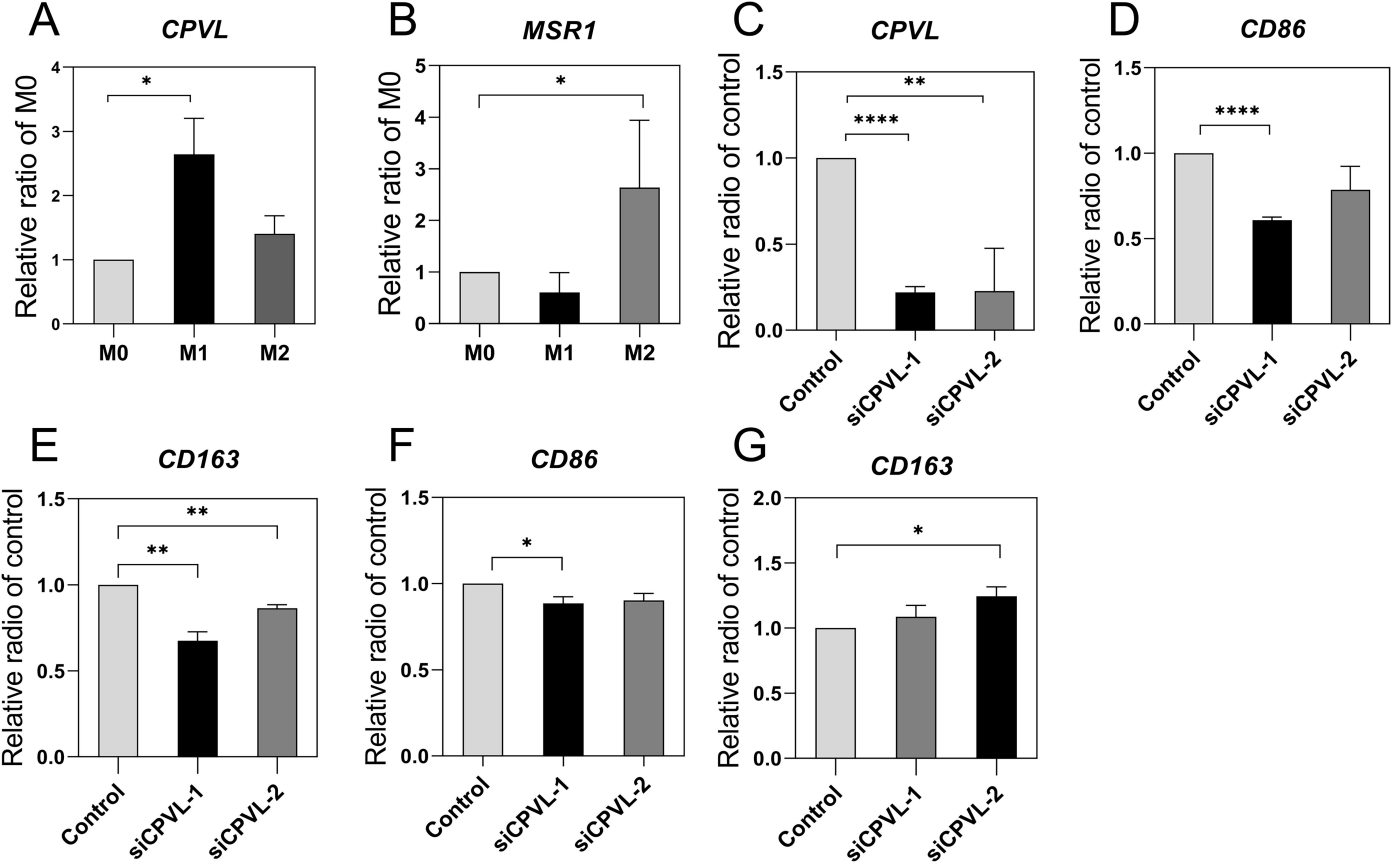
Effect of CPVL and MRS1 on macrophage polarization. PCR was performed for the target genes and cell marker to observe mRNA levels in macrophages with different polarization states, and the values were subjected to T-tests for statistical significance. (All data were expressed as mean ± SEM; n = 3, and each set of experiments was repeated three times). **(A, B)** CPVL and MSR1 expression in different polarization states. **(C-E)** CPVL, CD86, CD163 expression in M0 macrophages, cells were treated with PMA then transfected with siRNAs targeting CPVL. **(F, G)** CD86, CD163 expression in M1-polarized macrophages. *Indicates that the P value is less than 0.05. **Indicates that the P value is less than 0.01. ***Indicates that the P value is less than 0.001.

To clarify the role of CPVL and MSR1 in macrophage, we induced human macrophage polarization and measured mRNA expression of CPVL and MSR1 in this *in vitro* system (**Figures 6A, B**). CPVL expression is substantially upregulated in M1-like polarized macrophages, whereas M2-like macrophages show reduced CPVL expression (P). Conversely, MSR1 expression is substantially upregulated in M2-like polarized macrophages, while M1-like macrophages have reduced MSR1 expression (P). Our data were consistent with the aforementioned findings on patient prognosis, suggesting that CPVL and MSR1 influenced patient prognosis by regulating macrophage polarization states. Previous studies have shown that silencing MSR1 reduces macrophage polarization towards M2 in TME of gastric cancer (17), which agrees with our results. While the role of CPVL in macrophage polarization remains unclear. Initially, we knocked down CPVL to examine the expression levels of CD86, and CD163 in M0 macrophages to assess the polarization trend (**Figures 6C–E**). The results showed that CPVL silencing led to reduction in the expression of both M1 and M2 marker genes, with a more pronounced decrease in M1 marker genes compared to M2 markers. This shows that while CPVL affects both M1 and M2 macrophage polarization, its impact on M1 polarization is more substantial. Subsequently, we further induced the transfected macrophages to differentiate towards M1 polarization. The experimental results indicated that CPVL knockdown decreased the number of M1-polarized macrophage cells and increased the number of M2-polarized macrophage cells, which is consistent with our previous findings (**Figures 6F, G**). In summary, these findings suggests that while CPVL affects both M1 and M2 polarization, its effect on promoting M1 polarization is more significant.

**Figure 7:**
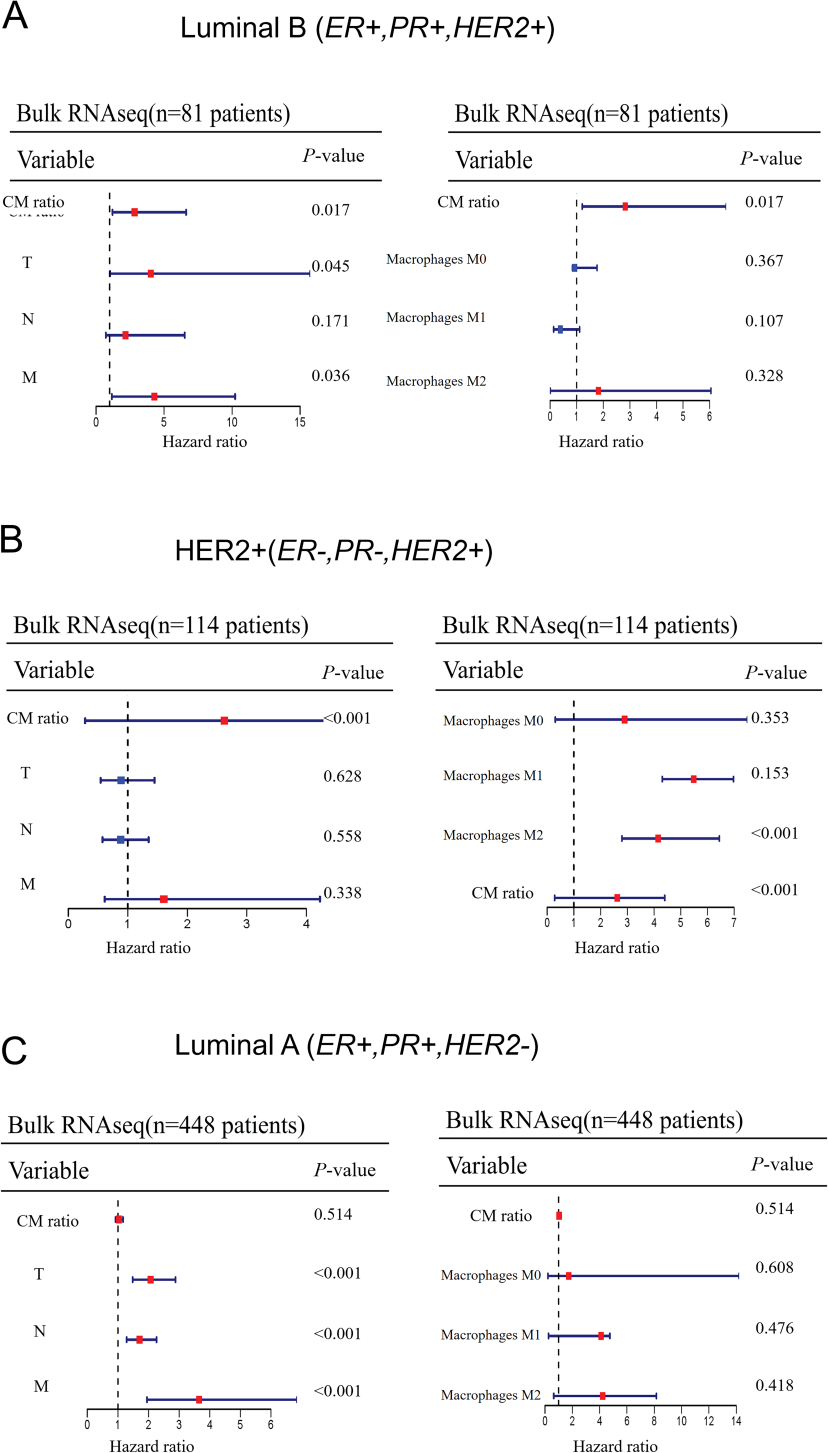
The extent to which CM ratio affects prognosis in other intrinsic molecular subtypes of breast cancer **(A-C)** A univariate Cox model was used to evaluate the impact of CM ratio, TNM stage, and macrophage infiltration on patient prognosis across other intrinsic molecular subtypes of breast cancer.

To investigate whether CPVL and MSR1 have prognostic significance in other intrinsic molecular subtypes of breast cancer, we analyzed their gene expression from 643 patients from the TCGA database. Using a univariate Cox model, we evaluated the impact of CM ratio, TNM stage, and macrophage polarization on patient prognosis across different breast cancer subtypes (**Figures 7A–C**). The CM ratio and macrophage polarization have no significant impact on prognosis in Luminal B (ER+, PR+, HER2+) and Luminal A (ER+, PR+, HER2-) subtypes. However, in the HER2-positive subtype (ER-, PR-, HER2+), both M2-like macrophage polarization and CM ratio are positively associated with poor patients’ prognosis. This suggests that, unlike in TNBC, CPVL and MSR1 may play a distinct role in HER2-positive subtypes, warranting further investigation.

## 4 Discussion

Recently, single-cell sequencing technology has advanced significantly, resulting in notable progress in TME investigation. However, single-cell sequencing data often lack clinical and prognostic information, making it challenging to analyze the role of key genes in cell subpopulations on disease prognosis and therapeutic potential, as is possible with bulk RNA sequencing. Although previous studies have integrated single-cell RNA sequencing with bulk RNA sequencing to clarify the relationship between cells and prognosis, these analyses were limited to a specific cell type (2–4, 48, 49) and lacked parallel comparisons across multiple cell populations. Therefore, in our study, we combined scRNA-seq with bulk RNA sequencing to highlight the impact of distinct cell types in the TME on patient outcomes. We found that immune cells were the most prognostically significant among the three major cell compartments, and TAMs showed the strongest correlation with TNBC prognosis in the immune cells.

TAMs have been shown to play a significant role in immune suppression within TME (50–52), making TAMs crucial for disease prognosis and treatment. Through Kaplan-Meier survival curve analysis of genes with prognostic significance in TAMs, we identified CPVL and MSR1 as key genes affecting prognosis in TNBC. While many genes have been identified to influence prognosis in TNBC patients by regulating macrophage polarization, such as OTUD5 (53), Sohlh2 (13), and COL5A1 (11), the impact of CPVL and MSR1 expression in TAMs on macrophage polarization in TNBC and their subsequent effect on patient prognosis remains undetermined.

Previous studies have shown that CPVL is positively correlated with M2-like macrophage polarization in gastric cancer, which leads to poor prognosis (15). Additionally, CPVL has been implicated in glioma, where its high expression in glioma cells is associated with poor prognosis and enhanced glioma cell proliferation (54). However, our survival analysis confirms that CPVL is associated with favorable prognosis in TNBC patients. Furthermore, our qRT-PCR analysis shows that CPVL expression is positively correlated with M1-like macrophages, but not with M2-like macrophages. This suggests that CPVL may play different roles in macrophages across various TME, providing new therapeutic insights for treating tumors in different contexts. Moreover, in TNBC patient macrophages, CPVL expression was elevated in both M1 and M2 polarized macrophages. Knockdown of CPVL in M0 macrophages suppressed the expression of both M1 and M2 markers, with a more pronounced inhibition of M1 markers. This indicates that CPVL mainly promotes M1 macrophage polarization in TNBC.

TAMs play a broad role in the microenvironment by interacting with various cell types through gene expression and cytokine secretion (55, 56), thereby influencing tumor progression and patient prognosis. In TNBC, the role of CPVL^+^ TAMs have not been previously studied. Our results indicated that CPVL is positively correlated with the prognosis of TNBC and M1-like macrophages. And TAMs expressing CPVL might interact with other cell types through multiple pathways. For instance, they may exert anti-tumor effects by inhibiting SPP1-CD44 interactions and promoting CXCL9-CXCR3 and C3-C3AR1 interactions, thereby engaging with monocytes, neutrophils, and T cells. At the same time, they may also promote tumor progression through interactions with fibroblasts, endothelial cells, and monocytes via GAS6-AXL and ANXA1-FPR1 signaling. Overall, CPVL plays a highly complicated role in the TNBC microenvironment.

Additionally, we observed that CPVL^+^ TAMs may achieve autocrine activation through the cytokines IL-18 and IFNγR1, which could enhance immune responses within the TME. Notably, CFD is one of the most reactive factors in osteoporosis triggered by chemotherapy or estrogen deficiency (57). In our investigation, we found a significant positive correlation between CFD gene expression and CPVL^+^ TAMs, offering a new perspecAt the same time, they may also promote tumor progression through interactions with fibroblasts, endothelial cells, and monocytes via GAS6-AXL and ANXA1-FPR1 signaling.tive for reducing osteoporosis risk during TNBC treatment.

MSR1 has been documented to undergo changes in both physiological and pathological processes associated with macrophages (17, 58), influencing conditions such as atherosclerosis and innate and adaptive immunity. MSR1 is also positively correlated with M2-like macrophage polarization (17, 59, 60). Though, no research has yet investigated the effect of MSR1 expression in TAMs of TNBC on patient prognosis. Our survival analysis revealed that MSR1 is strongly correlated with poor prognosis in TNBC patients, and qRT-PCR analysis confirmed its positive correlation with M2-like macrophages. We also found that the gene expression of CCL20, CCL2, and TREM2 was positively correlated with MSR1^+^ TAMs, suggesting that these factors may promote M2 macrophage polarization and suppress immune responses. TREM2 has been reported to promote macrophage polarization from M1 to M2 via the NF-κB/CXCL3 and JAK-STAT pathways (38, 61), and in lung cancer, TREM2^+^ macrophages were associated with a lack of NK cells and their dysfunction (62).

Beyond TNBC, we also explored the expression of CPVL and MSR1 in macrophages in other molecular subtypes of breast cancer using bulk RNA sequencing. In other subtypes, only the HER2+ subtype showed a substantial correlation between CM ratio and patient prognosis. Unlike TNBC, a higher CM ratio was related to poor prognosis in the HER2+ subtype. Therefore, our results also reflected tumor heterogeneity, demonstrating that immune response mechanisms varied across different tumor subtypes. This suggests that exploring the mechanism and role of CPVL and MSR1 in the HER2+ subtype represents a promising new research direction.

The TME is a complex ecosystem regulated by multiple interacting pro-tumor and anti-tumor signals, and we have not yet fully elucidated all the specific mechanisms by which CPVL and MSR1 function in TAMs. Due to the heterogeneity of macrophages, further studies in both cytological experiments and patient cohorts are needed to validate the prognostic value of CPVL and MSR1. Nevertheless, these preliminary findings underscore the prognostic significance of macrophages within the TME in TNBC, highlighting the critical role of CPVL and MSR1 expression in macrophages as key determinants of TNBC prognosis. This provides a theoretical foundation for the development of potential prognostic markers for TNBC patients, open new avenues for future TNBC research and suggests the therapeutic potential of targeting macrophages in TNBC treatment.

## 5 Conclusion

In conclusion, our work illuminates a prognostic profile of different cell subtypes in TNBC, and presents macrophages as the most prognostically significant cell type. Building on this, we also find the key genes within macrophages that influence TNBC prognosis — CPVL and MSR1. Although further studies on real-world TNBC cohorts are needed, this study provides new insights regarding future therapeutic approaches in TNBC patients.

## Data Availability

The datasets presented in this study can be found in online repositories. The names of the repository/repositories and accession number(s) can be found in the article/**Supplementary Material**.

## Supplementary material

Supplementary Figure 1 Global UMAP Dimensionality Reduction to Validate Gene Expression in Cells. **(A–E)**. UMAP analysis was used to assess the expression characteristics of the five selected genes across different cell types. (The five genes are primarily expressed in macrophages).

Supplementary Figure 2 Survival curve evaluation of seven genes **(A–G)**. Kaplan-Meier survival curves representing the probability of survival over time based on different expression levels of seven genes. The red curve indicates the high-expression group, while the blue curve represents the low-expression group. The x-axis shows time (in years), and the y-axis represents survival probability.
